# Spontaneous Transdiaphragmatic Intercostal Hernia in an Elderly Patient: A Rare Case Treated by Laparoscopic Repair

**DOI:** 10.7759/cureus.102679

**Published:** 2026-01-31

**Authors:** Jonathan Boukla, Maria Ruiz Patino, Martina Pezzullo, Patrizia Loi, Desislava Germanova

**Affiliations:** 1 Abdominal Surgery and Transplantation, Hôpital Universitaire de Bruxelles, Brussels, BEL; 2 Thoracic Surgery, Brussels University, Hospital Erasme, Brussels, BEL; 3 Digestive Radiology, Hôpital Universitaire de Bruxelles, Brussels, BEL; 4 Digestive Surgery and Transplantation, Hôpital Universitaire de Bruxelles, Brussels, BEL

**Keywords:** ct scan imaging follow-up, laparoscopic hernia repair, non traumatic hernia, radiological follow-up, surgical and clinical management, transdiaphragmatic intercostal hernia

## Abstract

Transdiaphragmatic intercostal hernia is a rare entity, with fewer than 50 cases reported in the literature. The majority are post-traumatic, secondary to blunt or penetrating thoracic injuries. Spontaneous forms are exceptional and often associated with risk factors such as obesity, chronic pulmonary disease, or long-term corticosteroid and immunosuppressive therapy. We present the case of a 71-year-old man with no history of trauma, who developed progressive dyspnea and subocclusive symptoms over several months. Imaging revealed a left transdiaphragmatic intercostal hernia between the seventh and eighth ribs, containing small bowel, colon, and omentum. The patient underwent laparoscopic repair with mesh reinforcement. Postoperative recovery was uneventful, with significant clinical improvement and no recurrence at eight-month follow-up. At that point, the patient reported localized neuropathic pain without radiological evidence of recurrence. This case highlights the rare occurrence of spontaneous transdiaphragmatic intercostal hernia, underlining the role of obesity and chronic immunosuppression as contributing factors, and emphasizes the diagnostic challenge of this condition as well as the feasibility and effectiveness of a laparoscopic mesh repair strategy.

## Introduction

Transdiaphragmatic intercostal hernia is an exceptionally rare condition, with fewer than 50 cases reported in the literature to date [1,2]. Most reported cases are post-traumatic, occurring after high-energy blunt thoracic trauma such as road traffic accidents [1-3], or following penetrating thoracic injuries, including stab wounds and gunshot wounds [4,5]. In a smaller proportion of cases, underlying pulmonary pathology, particularly chronic obstructive pulmonary disease, has been identified as a contributing factor [6]. Spontaneous forms, occurring in the absence of trauma or obvious pulmonary disease, remain exceptional [7,8]. 

Spontaneous transdiaphragmatic intercostal hernias are clinically challenging, as symptoms are often progressive and non-specific, which may result in delayed diagnosis. Patients may present with respiratory complaints, chest or abdominal pain, or digestive symptoms, especially in the absence of a traumatic context. 

Clinical presentation is variable, ranging from mild or progressive dyspnea to digestive symptoms related to herniation of abdominal viscera. Computed tomography is the imaging modality of choice, as it allows precise identification of the diaphragmatic and intercostal defect, characterization of hernia contents, and evaluation of associated thoracic or abdominal complications. CT imaging is also essential for surgical planning. 

We report here the case of a 71-year-old man with no history of trauma, who developed a progressive transdiaphragmatic intercostal hernia. Predisposing factors included obesity and longstanding rheumatoid arthritis treated with chronic corticosteroid and immunosuppressive therapy. This case is notable for its atypical non-traumatic presentation and for its successful management using a laparoscopic approach, with a favorable outcome.

## Case presentation

Patient presentation

A 71-year-old man was referred to surgical consultation for progressive dyspnea and intermittent subocclusive symptoms, which had begun approximately one year prior to diagnosis and had gradually worsened over time. The patient also reported associated abdominal pain and pyrosis of similar duration. 

His past medical history included obesity (BMI 35.9), chronic renal failure, atrial fibrillation with a pacemaker, and longstanding rheumatoid arthritis treated with methylprednisolone and immunosuppressive therapy (methotrexate 25 mg/week and filgotinib 200 mg/day, later switched to sarilumab). He had required a prolonged intensive care unit stay in 2022 for respiratory failure secondary to infectious pneumonia. No history of thoracic or abdominal trauma was reported, and the patient denied any episodes of severe coughing, vomiting, or other Valsalva-type events preceding the onset of symptoms.

Diagnostic assessment

**Figure 1:**
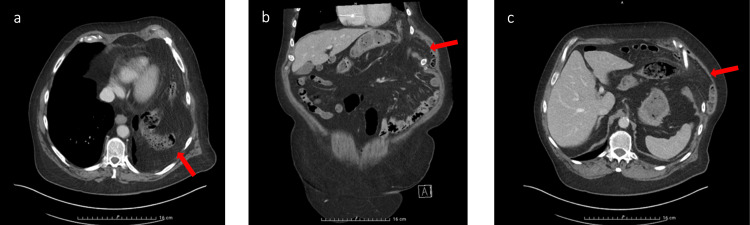
Left transdiaphragmatic intercostal hernia: preoperative thoracoabdominal CT scan imaging. a. Thoracic sagittal view b. Frontal thoracoabdominal view c. Abdominal sagittal view

Upper gastrointestinal endoscopy revealed gastritis without evidence of *Helicobacter pylori* infection. A thoraco-abdominal computed tomography scan demonstrated a left intercostal transdiaphragmatic hernia between the seventh and eighth ribs, with an estimated defect size of 41 × 55 mm, containing small bowel, colon, and omentum. No hernia sac was identified (Figure 1). There were no radiological signs of bowel strangulation or ischemia, including bowel wall thickening, reduced enhancement, pneumatosis, or mesenteric congestion.

Surgical management

**Figure 2:**
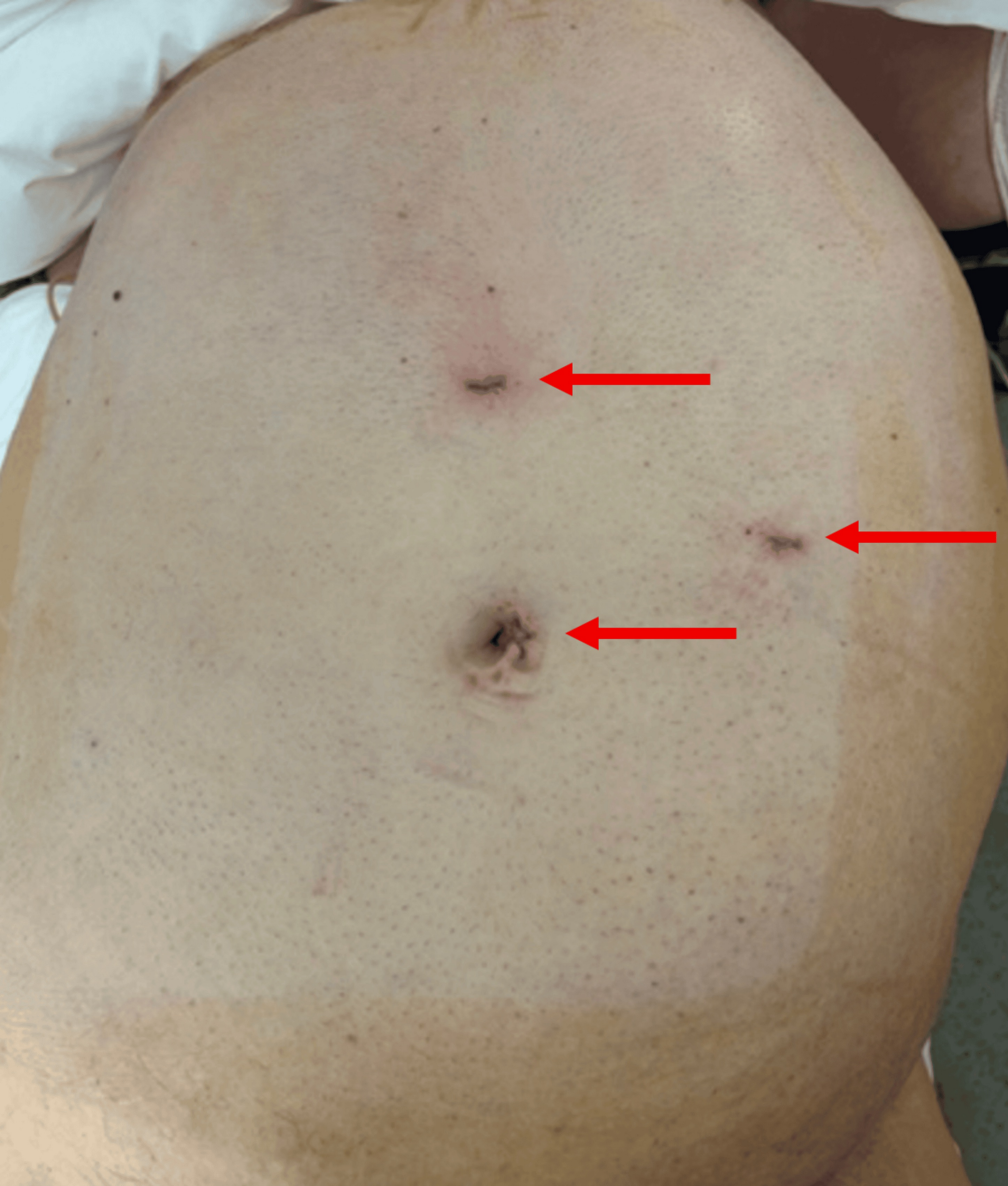
Port placement. A 12-mm supra-umbilical port, one 5-mm epigastric port, and one 5-mm port in the left hypochondrium

A laparoscopic repair was performed under general anesthesia, with the patient in a supine position and legs apart. Pneumoperitoneum was established using a Veress needle at 12 mmHg. Three trocars were inserted: one 12-mm supra-umbilical port, one 5-mm epigastric port, and one 5-mm port in the left hypochondrium (Figure 2). 

Exploration revealed a transdiaphragmatic intercostal hernia containing small bowel, colon, and omentum. Reduction was achieved easily, without adhesions. The abdominal cavity communicated directly with the thoracic cavity through the hernia defect, without interposition of a hernia sac. The thoracic cavity was free of adhesions. 

After reduction, the intra-abdominal pressure was lowered to 8 mmHg, and the defect was closed using two hemi-continuous V-Lok sutures. Given the size of the defect (41 × 55 mm), a wide mesh overlap was deemed necessary, and a 15-cm circular Parietex™ (Medtronic, Minneapolis, USA) composite mesh was placed to ensure adequate coverage and reduce the risk of recurrence. Fixation was achieved using a combined technique, with lateral fixation using absorbable tackers (AbsorbaTack™ [Medtronic, Minneapolis, USA]) and medial fixation using interrupted 3-0 polypropylene sutures, in order to provide secure anchorage while limiting the risk of mesh displacement. 

A pulmonary recruitment (Valsalva) maneuver was performed prior to definitive closure to minimize the risk of postoperative pneumothorax. Intraoperative thoracic surgical consultation was obtained, confirming that thoracic drainage was not required. The estimated operative time was 45 minutes, with no significant intraoperative blood loss.

Postoperative course

**Figure 3:**
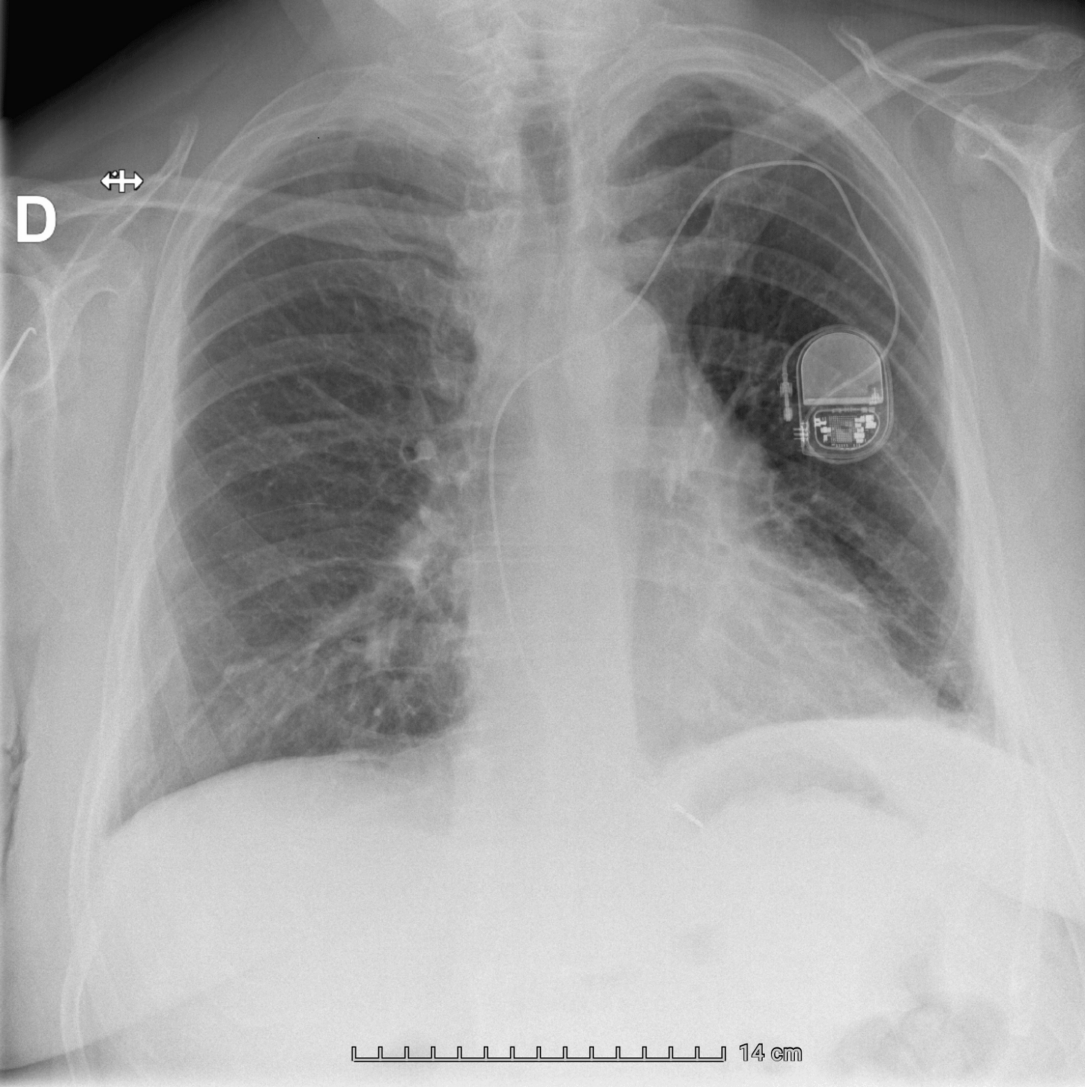
Control chest X-ray on postoperative day one.

The postoperative course was uneventful. The patient reported mild parietal pain, which was adequately controlled with step I analgesics. Chest radiographs obtained on postoperative day (POD) 0 and POD 1 excluded pneumothorax (Figure 3). Early mobilization was initiated on POD 1, and the patient was discharged on POD 1 with oral analgesics and instructions to avoid heavy lifting (>3-4 kg).

At the one-month follow-up, a control CT scan showed no evidence of recurrence, and the patient reported marked improvement in both respiratory and digestive symptoms.

**Figure 4:**
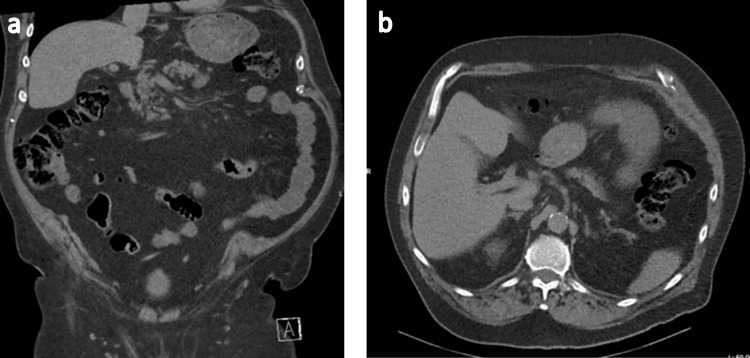
Control CT scan eight months after surgery. No recurrence was observed in frontal view (a) nor in sagittal view (b).

At the eight-month follow-up, the patient was reviewed in outpatient consultation. He reported localized exertional parietal pain in the area corresponding to the previous hernia site, described as intermittent “electric discharges,” without associated dyspnea or digestive symptoms. A repeat CT scan confirmed the absence of recurrence (Figure 4). Neuropathic pain related to intercostal nerve involvement was suspected. Referral to a pain management clinic was proposed; however, the patient ultimately declined further evaluation, as symptoms were considered tolerable. No specific neuropathic pharmacological treatment was initiated.

## Discussion

Transdiaphragmatic intercostal hernia is a very uncommon condition, with fewer than 50 cases reported in the literature [2]. The majority of reported cases are post-traumatic, following high-velocity blunt trauma or penetrating injury [2-5]. Other etiologies include underlying pulmonary pathology, notably chronic obstructive pulmonary disease, in which repeated increases in intrathoracic pressure may contribute to progressive weakening of the diaphragm and intercostal spaces [6]. Rare spontaneous cases have also been described [7,8]. In our patient, no traumatic event was identified. Two risk factors likely contributed. Obesity is known to increase intra-abdominal pressure and has been associated with the development of abdominal wall hernias [9]. In addition, longstanding rheumatoid arthritis treated with chronic corticosteroid and immunosuppressive therapy may predispose to progressive muscular and tissue fragility, potentially affecting diaphragmatic and intercostal structures [10]. Although the exact pathophysiological mechanisms remain incompletely understood, this combination of increased mechanical stress and reduced tissue resistance may facilitate hernia formation in the absence of trauma.

**Figure 5:**
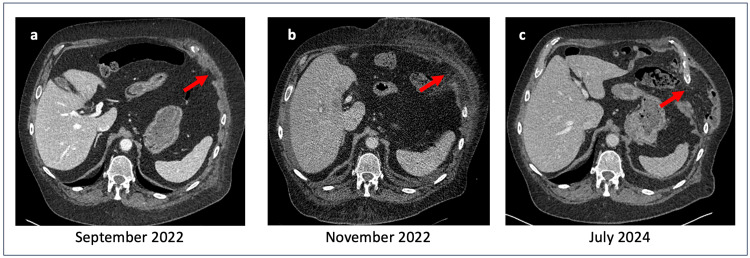
Sagittal view of thoracoabdominal CT scan showing the evolution of the left transdiaphragmatic intercostal hernia over time.

**Figure 6:**
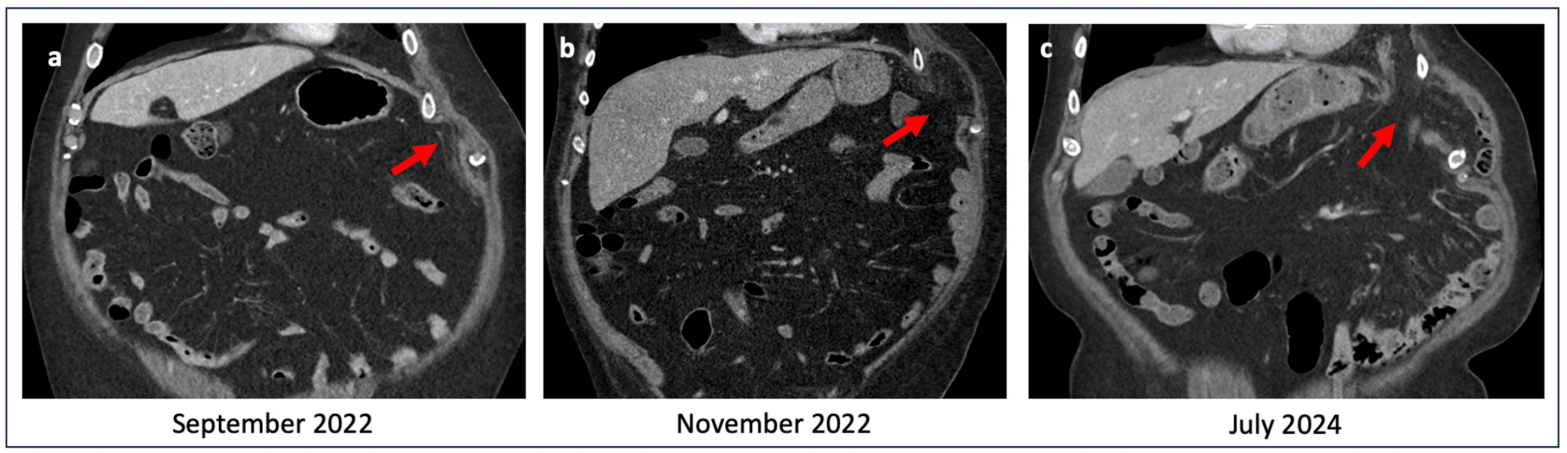
Frontal view of thoracoabdominal CT scan evolution of the left transdiaphragmatic intercostal hernia over time.

The natural history of transdiaphragmatic intercostal hernias may be acute or progressive, with some cases developing over several years [11]. In this patient, CT imaging from 2022 had already demonstrated early signs of an intercostal hernia associated with local inflammatory changes. Over time, the hernia progressed through the diaphragm, supporting a gradual evolution rather than an acute rupture (Figures 5, 6). Negative intrathoracic pressure during respiration has been proposed as a contributing factor to the progressive enlargement of diaphragmatic defects [12]. However, due to the rarity of this condition, it remains unclear whether negative intrathoracic pressure, increased intra-abdominal pressure, or a combination of both plays the predominant role in hernia formation.

Surgical repair remains the only curative treatment [13,14]. Both open approaches (thoracotomy or laparotomy) and minimally invasive techniques have been described. In selected cases, laparoscopy may be preferable to thoracotomy, as it allows excellent visualization of the diaphragmatic defect, facilitates safe reduction of abdominal viscera, and enables mesh reinforcement with reduced postoperative pain and faster recovery. These advantages may be particularly relevant in elderly or comorbid patients [2]. For large defects, prosthetic reinforcement with mesh is recommended to reduce the risk of recurrence [4,15]. In our case, primary closure was performed using V-Lok sutures and reinforced with a Parietex™ mesh. No thoracic drain was required, as no pneumothorax occurred, consistent with previous reports [15]. 

The optimal method of mesh fixation remains debated. In our patient, lateral fixation was achieved using tackers and medial fixation with interrupted sutures, providing secure fixation without displacement or recurrence. This approach differs from reports suggesting a potential risk of mesh migration when tackers alone are used [4]. 

Another interesting feature in this case was the absence of a hernia sac. This situation is more frequently described in acute or traumatic hernias, whereas progressive or congenital hernias generally involve a sac enclosing the herniated viscera [16]. In our patient, imaging from 2022 suggested a progressive mechanism, but the absence of a sac at surgery may indicate a superimposed acute event. The current literature does not clearly distinguish outcomes between patients with or without a sac, as this feature is inconsistently reported. 

Most common early postoperative complications include pulmonary complications (pneumothorax, hemothorax, pulmonary infection), abdominal complications (ileus, bowel obstruction), and, as a late complication, recurrence and complications related to the mesh (migration, erosion, infection) [2,3].

## Conclusions

Spontaneous transdiaphragmatic intercostal hernia is an exceptionally rare entity, with fewer than 50 cases reported in the literature. In this patient, the hernia developed progressively over several years without any history of trauma, illustrating that non-traumatic etiologies can occur. This case highlights the need to maintain a high index of suspicion in obese or immunosuppressed patients presenting with atypical and progressive thoracoabdominal symptoms, even in the absence of trauma. Early diagnosis and timely laparoscopic repair with mesh reinforcement can lead to favorable short- and mid-term outcomes, with a low risk of recurrence.
